# Study on Representation Invariances of CNNs and Human Visual Information Processing Based on Data Augmentation

**DOI:** 10.3390/brainsci10090602

**Published:** 2020-09-02

**Authors:** Yibo Cui, Chi Zhang, Kai Qiao, Linyuan Wang, Bin Yan, Li Tong

**Affiliations:** Henan Key Laboratory of Imaging and Intelligent Processing, PLA Strategic Support Force Information Engineering University, Zhengzhou 450001, China; blackchill@163.com (Y.C.); zcboluo@hotmail.com (C.Z.); qiaokai1992@gmail.com (K.Q.); wanglinyuanwly@163.com (L.W.); ybspace@hotmail.com (B.Y.)

**Keywords:** representation invariance, CNNs, human visual information processing, data augmentation, fMRI visual encoding model

## Abstract

Representation invariance plays a significant role in the performance of deep convolutional neural networks (CNNs) and human visual information processing in various complicated image-based tasks. However, there has been abounding confusion concerning the representation invariance mechanisms of the two sophisticated systems. To investigate their relationship under common conditions, we proposed a representation invariance analysis approach based on data augmentation technology. Firstly, the original image library was expanded by data augmentation. The representation invariances of CNNs and the ventral visual stream were then studied by comparing the similarities of the corresponding layer features of CNNs and the prediction performance of visual encoding models based on functional magnetic resonance imaging (fMRI) before and after data augmentation. Our experimental results suggest that the architecture of CNNs, combinations of convolutional and fully-connected layers, developed representation invariance of CNNs. Remarkably, we found representation invariance belongs to all successive stages of the ventral visual stream. Hence, the internal correlation between CNNs and the human visual system in representation invariance was revealed. Our study promotes the advancement of invariant representation of computer vision and deeper comprehension of the representation invariance mechanism of human visual information processing.

## 1. Introduction

Deep convolutional neural networks (CNNs) have not only obtained success in the computer vision domain for their unprecedented applications, but have also attracted the attention of workers in the field of psychology and neuroscience. Recently, considerable literature has indicated that CNNs have become an important tool for research in neuroscience [1,2,3,4,5,6,7]. Using CNNs for modeling the human brain visual system based on functional magnetic resonance imaging (fMRI) is becoming a bridge connecting artificial intelligence (AI) and human intelligence. The structure of CNNs was initially inspired by human visual information processing, which leads to some natural similarities between CNNs and the human visual system [8,9,10]. One of the most significant current discussions in AI and computational neuroscience is regarding the similarities and differences of information processing mechanisms between CNNs and the human brain visual system [10,11,12,13,14]. For visual information processing, CNNs and the human brain visual system have shown some invariances [14,15,16], which are the objects that may still be classified or identified quickly after translation, rotation, and scaling transformation. Further study of the similarities and differences of invariance of CNNs and the human brain visual system can help us continue to gain understanding of the human brain visual system, and also to promote the further development of computer vision.

Although CNNs have been the go-to architecture for image-based tasks such as detection, segmentation, classification, and so on, CNNs are still a “black box” for all researchers [10,17,18]. However, considerable literature has accumulated about the visual information processing mechanisms of similar invariant representation recently. It was generally thought that CNNs had the capacity of invariant representation for several factors [19,20,21]. One undoubted factor is the big dataset. The deep network can learn a certain pattern of a class of objects in various complex scenarios from a large number of examples, implementing invariant representation for the object category. Therefore, CNNs could improve the capacity of translational invariance through data augmentation or feature augmentation [22,23]. Another factor is the specific architecture of CNNs [19,20,24,25,26,27]. The representation invariance of CNNs lies in the operation of weight sharing and pooling in local receptive fields of convolution layers. Local receptive fields with shared weights can catch invariant elemental features despite variations in the positions of conspicuous features caused by small local shifts or distortions of the input images. Furthermore, pooling operations, comparable to subsampling, reduce the resolution of the feature maps in each layer, which drives more robust position transformation invariance [19,20]. For other complex transformations such as rotation in-plane, rotation in-depth, and scale, however, there remains no specific invariance mechanism. Therefore, there has been discussion whether the combination of convolution layers and fully connected layers is an important factor for the transform-invariant property of CNNs.

Due to CNNs derived from the hierarchical ventral visual stream [28,29], it is commonly demonstrated that exploring the representation invariance in the visual information processing process of the human brain is not only practical for understanding the brain but also valuable for analyzing the transform-invariant property of CNNs. Several empirical studies have examined whether neurons have lower invariance for progressively perplexing stimulus features in each successive region of interest (ROI) of the ventral visual stream [30,31,32,33,34,35]. Other literature indicates that neurons become more invariant to transformations, such as image shifts, distortions, translation, scaling, and rotation in plane [36,37,38,39]. Gross proposed that the responses of neurons in senior visual areas are simultaneously more selective for perplexing stimulus features [40]. Specifically, the selectivity of V4 neurons for contour curvature was studied in subsequent studies. El-Shamayleh et al. described that most V4 neurons (73%) were size-invariant to objects, and encoded stimulus in a position-independent manner in the receptive field (RF) [41], as contrasted to another study using form stimulus characterized by combinations of line ingredients, which found that many neurons in V4 depended on the position of the stimulus [42]. Sharpee et al. suggested that many V4 neurons were limitedly invariant, and relatively few neurons have high invariance [43]. In the inferior temporal cortex (IT), a more high-level region, neurons have higher invariance than V4 neurons, which is inversely related to conjunction selectivity [44]. In general, a high degree of invariance is negatively correlated with the degree of selectivity [36,37,45], although another study has found neurons with strong invariance and strong selectivity together [46]. However, most previously published studies are limited to high-level ROIs rather than the whole process of the ventral visual system.

In summary, the studies for CNNs and human visual information processing powerfully promoted each other. However, the mechanism of representation invariance has been subject to confusion in both CNNs and human visual information processing. There is not much literature studying the two systems together to investigate their relationship under common conditions.

To solve the above problems, this paper mainly studies the representation invariance mechanisms of CNNs and the human brain visual system and their relationship. We proposed a representation invariance analysis approach based on data augmentation technology, a strategy that increases both the amount and diversity of data by randomly “augmenting” it. In the image domain, common augmentations include translating, scaling, cropping, flipping, and so on. Firstly, the original image library was expanded by data augmentation technology. Then, a pre-trained convolutional neural network (CNN), Alexnet [22], was used to extract features of original images and corresponding augmented images. Similarities of the features in corresponding intermediate layers were compared to study the role of CNNs network structure for invariant representation. To study the representation invariance of ROIs of the ventral visual stream, three aspects as follows were studied based on the fMRI visual encoding model: (1) whether encoding models select more invariant features after data augmentation; (2) the impact of data augmentation on the encoding performance for each ROI; (3) the percentages of the voxels accurately predicted in each ROI before and after data augmentation. Finally, we investigated the similarities and differences of the representation invariance mechanisms of CNNs and the human brain visual system. Experimental results showed that the collective effect of convolution layers and full connection layers promoted representation invariance of CNNs. Transformation invariance exists in the whole process of the ventral visual stream, and there is a tendency that transformation invariance is increasingly strong along successive stages of the human information processing process. Therefore, it was remarkably found that there was an internal correlation in representation invariances of CNNs and the human visual information processing process. The study will not only be helpful for deeply understanding the invariant representation mechanisms of the brain visual system and CNNs, but also promote the development of the invariant representation of computer vision.

## 2. Materials and Methods

### 2.1. The fMRI Data

The stimuli images and corresponding brain responses dataset used in this study were primitively published in previous studies [47,48], which can be downloaded from http://crcns.org/data-sets/vc/vim-1. Therefore, only a brief overview of the fMRI experiment is presented here, and more details can be found in those studies. The estimated blood oxygen level-dependent (BOLD) responses of two subjects (S1 and S2) were collected using a 4T INOVA MR scanner (Varian, Inc., Palo Alto, CA, USA). Eighteen coronal slices were acquired covering the occipital cortex (slice thickness 2.25 mm, slice gap 0.25 mm, field of view 128 × 128 ${\mathrm{mm}}^{2}$). fMRI data were acquired using a gradient-echo EPI pulse sequence (matrix size 64 × 64, TR 1 s, TE 28 ms, flip angle 20°, spatial resolution 2 × 2 × 2.5 ${\mathrm{mm}}^{3}$). For each subject, five sessions of fMRI data were acquired when subjects were displayed with experimental stimuli, which consisted of grayscale natural images (20 × 20°) drawn randomly from different photographic collections. Subjects fixated on a central white square (0.2 × 0.2°). Stimuli were flashed at 200 ms intervals for 1 s followed by 3 s of gray background in successive 4 s trials. Training and test data were acquired in the same scan sessions. The training set contained 1750 images, and each of them was repeated two times. Correspondingly, the testing set contained 120 images, and each of them was repeated 13 times. The BOLD responses in V1, V2, V3, V4, and lateral occipital (LO) regions were selected for further study. Figures in this study refer to the data from subject 1.

### 2.2. Invariant Representation and Data Augment Technology

**Figure 1:**
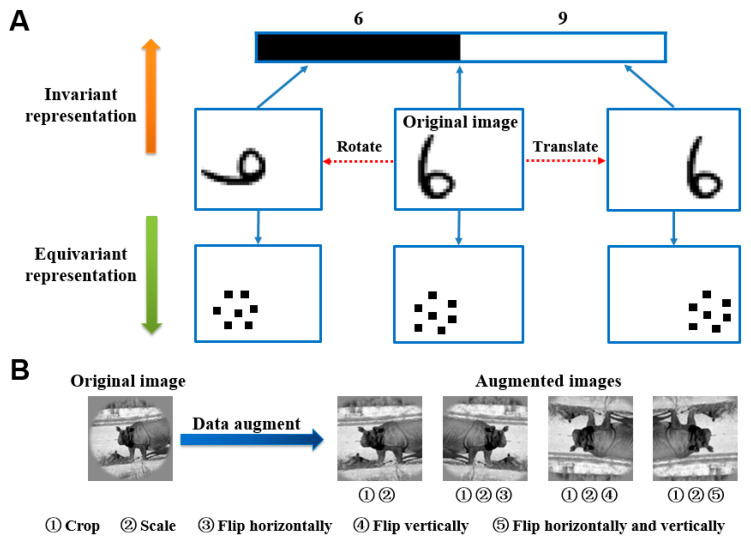
Feature representation and data augmentation technology. (**A**) Invariant representation and equivariant representation. The original image is rotated and translated to generate new images. The upward direction is an invariant representation, mostly for senior semantic features such as category. For example, “6” will be classified as 6 or 9. Conversely, the upward direction is an equivariant representation, mostly for low-level features, such as position and texture. (**B**) Data augmentation technology. The data augmentation image library (right) is obtained by cropping, scaling, and flipping sequentially (left).

In image classification and other visual tasks, CNNs and brain visual systems were invariant to some image transformations, owing to their invariant feature representation capacities [29]. Invariant feature representation means that the image features in CNNs and the human brain visual system change rarely due to image translation, rotation, perspective transformation, scale transformation, or other transformations during image representation. On the contrary, it is an equivariant feature representation. Figure 1A represents a schematic view of invariant feature representation and equivariant feature representation. Invariant representation generally appears in image senior semantic features extraction, and then equivariant representation is more related to image position, texture, and other detail features.

Data augmentation technology was originally proposed to countervail the shortage of training data. However, data augmentation technology was used from another perspective herein, which is studying the mechanisms of CNNs and fMRI visual information processing for invariant representation. For each image in the original training image library, 10 similar images were generated through the following steps (see Figure 1B): firstly, an image was cropped with 10 random sizes based on preserving the main body of the object in the image; then, the ten obtained images were enlarged to the same size as the original image; finally, the ten images were randomly flipped in one of four ways including horizontally, vertically, both, and neither. In this way, the augmented training image library was obtained. The original training library had 1750 images, and the augmented training library had 17,500 images. To study the representation invariance of CNNs, the feature similarities of each two corresponding intermediate layers were compared after CNNs were trained by the original dataset and augmented dataset respectively. To study the representation invariance of the human brain visual system, prediction responses of each ROI in the ventral visual stream before and after data augmentation were compared, which were generated by visual encoding models based on fMRI.

### 2.3. The Analytical Method for the Representation Invariance of CNNs

The role of the network architecture for the representation invariance of CNNs is mainly studied in this paper. Unlike the human brain visual system, the network structure and parameters of CNNs are known. Hence, the intermediate layer features of CNNs can be directly analyzed for similarities and differences between the CNN’s understanding of original images and augmented images. Alexnet, one of the CNNs, is studied herein. The cosine distance was used to metric the similarity of corresponding intermediate layer features, which was obtained after images were input into Alexnet. See Equation (1).

(1) $$D_{cos}=\frac{F_{l}\left(I_{o}\right)F_{l}\left(I_{A}\right)}{|\left|F_{l}\left(I_{o}\right)\right|||\left|F_{l}\left(I_{A}\right)\right||}$$

where *D_cos_* is the cosine distance, and *I_o_* represents an original image, and *F_l_* (*I_o_*) is the feature vector of *I_o_* extracted from the layer *l* of Alexnet, and *I_A_* represents the corresponding augmented image, and *F_l_* (*I_A_*) is the feature vector of *I_A_* extracted from the layer *l* of Alexnet. By statistical analysis of the cosine distance, the similarity of the corresponding intermediate layer features was studied, further studying the invariant representation of Alexnet.

### 2.4. Visual Encoding Models Based on fMRI

By simulating the information processing mode of the brain visual system, a visual encoding model was constructed to predict the visual cortex responses to different external visual stimuli. This technology was achieved by designing a computable mathematical model based on the biologic mechanisms of visual information processing based on fMRI. Therefore, the visual encoding model can be identified as a simulation brain visual system.

**Figure 2:**
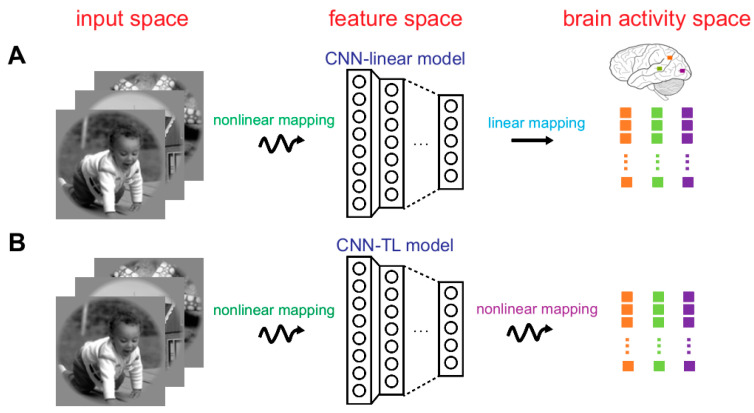
Visual encoding models. (**A**) The CNN-linear model. The first step is a nonlinear mapping that a pre-trained CNN (i.e., AlexNet) represents image features to construct the feature space. The second step is the linear mapping from the feature space to the brain activity space. In the brain activity space, different colored dots represent the responses of different voxels. Each dot represents the response of a voxel to one image. (**B**) The CNN-TL model. The first step is the same as that of CNN-linear model, but the second step is the nonlinear mapping, achieved by transfer learning.

The following two encoding models with marvelous prediction accuracy were used to study the representation invariance of the human brain visual system herein. For each picture, the actual response of the voxel was obtained by fMRI, and the predicted response was generated by visual encoding models. For a voxel, the prediction accuracy was defined as the Pearson correlation between the actual and the predicted responses across all 120 images in the test set [7]. The first model was the CNN-linear encoding model (see Figure 2A) [2]. In this model, pre-trained Alexnet was used as a nonlinear feature extractor to constitute the feature space firstly. Then from the feature space to brain activity space, multiple linear regression models were constructed for each voxel of the ROIs (V1, V2, V3, V4, and LO). These models were solved by a sparse linear regression optimization algorithm. Based on eight layers of Alexnet, eight encoding models were constructed for each voxel on the training dataset. By using the test dataset, the finest model with the best prediction performance was decided. The finest layer was selected simultaneously. The second model was the CNN-TL encoding model (see Figure 2B) [7]. Rarely different from the first model, nonlinear mapping was designed from the feature space to the brain activity space. To implement the nonlinear mapping, two fully connected layers were arranged after each layer of pre-trained Alexnet. The approach to select the best layer was the same as the first model. Replacement test experiments showed that if the encoding accuracy of a voxel was higher than 0.27 (*p* < 0.001) for any encoding model, the voxel could be accurately predicted. Other details about the above two encoding models can be obtained in published work.

### 2.5. The Analytical Method for Representation Invariance of Human Visual Information Processing

**Figure 3:**
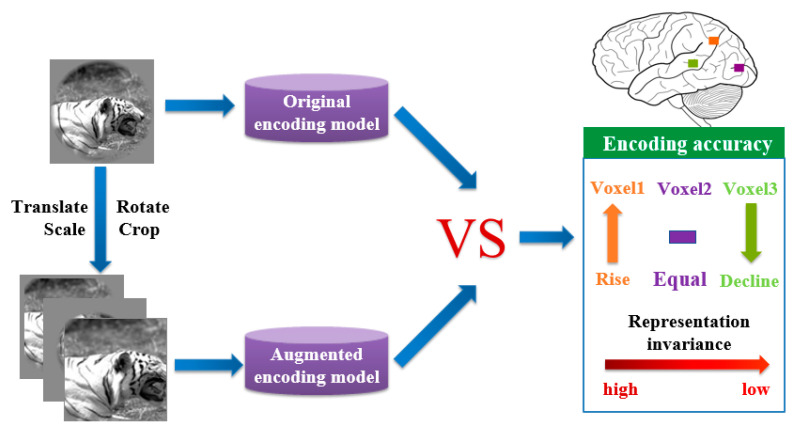
An analysis method of human visual invariance based on the fMRI visual encoding model. The original encoding model and augmented encoding model are trained by the original image library and data augmentation image library respectively. Prediction accuracies of each voxel predicted by the two models were compared. If the prediction accuracies of voxel 1 (orange), voxel 2 (purple), voxel 3 (green) rise, remain unchanged, and decline, respectively, there will be a decreasing tendency of representation invariance from voxel 1 to voxel 3.

Because it is burdensome to acquire the responses of the brain visual cortex for each original image and augmented image, the representation invariance of the human brain visual system was studied based on the above visual encoding models in this paper. Notably, it was hypothesized that similar responses are generated from voxels with representation invariance for an original image and the corresponding augmented image. Based on this hypothesis, augmented images were labeled the same responses of the corresponding images. Augmented images and their responses composed an augmented dataset. By using an augmented dataset, the above two original visual encoding models were trained to obtain augmented visual encoding models. Original visual encoding models and augmented visual encoding models predicted responses of each voxel for test images relatively. By comparing two corresponding responses, voxels better and worse predicted by the augmented model could be distinguished (see Figure 3). A rational reason is that the former is more sensitive to invariant features and the latter is more sensitive to equivalent features. Therefore, invariant representation along the ventral visual pathway can be studied through investigating the differences of changes in the prediction accuracy for each voxel in those ROIs, and further study on the representation invariance of the human brain visual system.

## 3. Results

### 3.1. The Representation Invariance of CNNs

**Figure 4:**
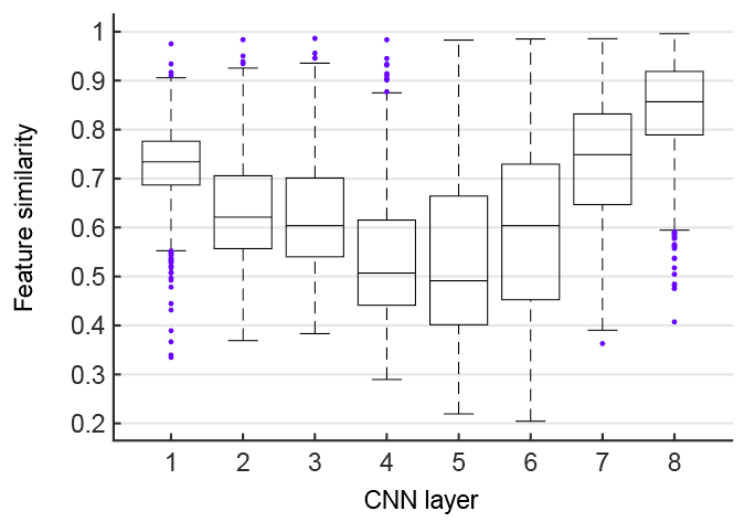
Features similarities. Each box shows feature similarities of a pair of corresponding CNN layers before and after data augmentation.

As shown in Figure 4, the feature similarities between the corresponding layers of the CNN before and after data augmentation is displayed using boxplots, which are defined as the average feature similarities over all possible pairs of the original images and augmented images. It can be found that the feature similarities of the first five corresponding layers of the CNN have a downward trend overall (Mann–Kendall test, *p* < 0.05). However, starting from the sixth layer, the feature similarities of the corresponding layers of CNN have shown an upward trend (Mann–Kendall test, *p* < 0.05), and the highest similarity among all layers is reached on the last layer. This phenomenon is seemingly not a coincidence, because the first five layers are the convolutional layers, and the last three layers are the fully connected layers exactly. On the one hand, the feature similarities of the convolutional layer show a gradual downward trend after data augmentation, and are only about 50% at the fourth and fifth layers. Therefore, the convolutional layer feature maps in CNNs are decreasingly invariant to transformations of the input images. In contrast, the equivariance of features is more obvious with the superposition of the number of convolutional layers. On the other hand, the similarity of fully connected layer features shows a gradual upward trend after data augmentation, indicating that the fully connected layers play a role in feature integration. The fully connected layer features have more invariance after being processed by the CNN fully connected layers, which improve the robustness of the CNN in image classification tasks. High feature similarity has been obtained between the last corresponding layers, indicating that data augmentation images can be recognized by the CNN well and robustly.

### 3.2. The Representation Invariance of Human Visual Information Processing

The encoding performances of the fMRI visual encoding models were compared after data augmentation to study the human brain visual representation invariance. As mentioned in Materials and Methods, two encoding models, the CNN-linear model and the CNN-TL model, were used for research. The following three questions were specifically studied: (1) whether the augmented encoding models select more invariant features was verified by comparing the tendencies of the encoding model and the augmented model for the selection of CNN layer features; (2) the impact of data augmentation on the encoding performance for each ROI was evaluated overall by comparing the prediction accuracies of the encoding model and the augmented model for voxel responses; (3) the regularity of invariant feature representation in each ROI was analyzed by firstly counting the voxels that can be accurately predicted in each visual cortex ROI and secondly calculating the proportion of the voxels that can be most accurately encoded by the encoding model and the augmented model.

#### 3.2.1. Whether the Augmented Encoding Models Select More Invariant Features

**Figure 5:**
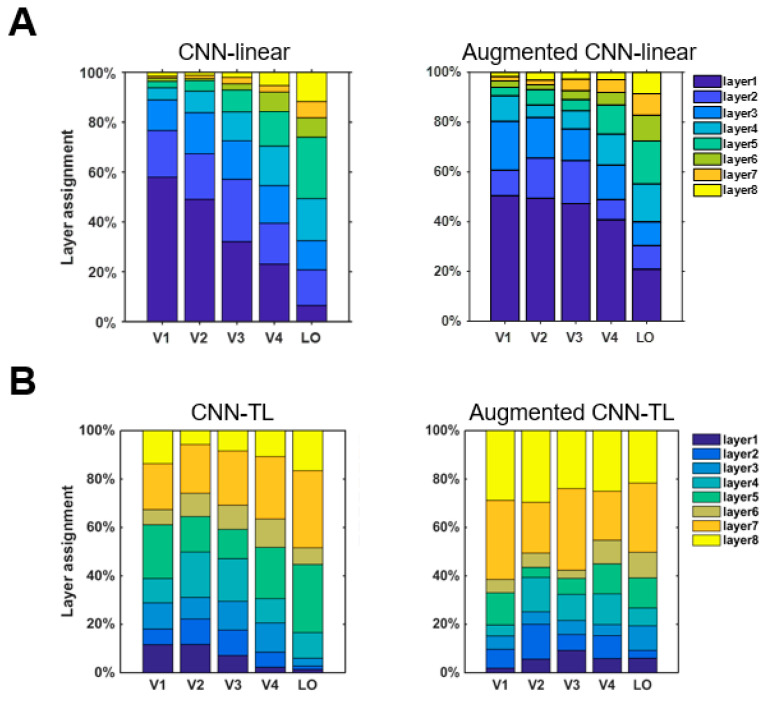
CNN layer preferences of the ventral visual stream in visual encoding models. (**A**) CNN layer preferences of the ventral visual stream in the CNN-linear model and augmented CNN-linear model. The CNN layer preferences of each ROI distribute in a single column. The contribution to the mean prediction accuracy of all voxels in that ROI is shown by colored bars within each column. (**B**) CNN layer preferences of the ventral visual stream in the CNN-TL model and augmented CNN-linear model.

In the construction of the encoding model, an optimal encoding model was selected from the eight middle layers of the CNN as the final encoding model for each voxel. For accurately predicted voxels in each visual cortex ROI, which layer the best encoding model was from was calculated to analyze which intermediate layer features were selected by the encoding model. To verify whether the augmented encoding models prefer to choose more invariant features, we compared the tendencies of the encoding model and the augmented model for the selection of intermediate layer features. The results of CNN-linear and CNN-TL models are shown in Figure 5A,B respectively. According to the analysis results of the invariant features of CNN in Section 3.1, the features of CNN layers could be divided into two groups according to the features invariance. The group with stronger invariance is the features of layers 1, 2, 7, and 8, and the group with weaker invariance is the features of layers 3, 4, 5, 6. The results in Figure 5A show that the percentages of using stronger invariant features when the CNN-linear model encoded voxels of each ROI before data augment were 78.9%, 69.8%, 61.6%, 47.3%, and 38.9%. The percentages for the augmented CNN-linear model were 64.1%, 70.6%, 71.8%, 56.9% and 47.6%. The percentages of all ROIs improved except V1. The results in Figure 5B show that the percentages of using stronger invariant features when the CNN-TL model encoded voxels of each ROI before data augmentation were 50.6%, 48.1%, 48.4%, 44.9%, and 51.1%. The percentages for the augmented CNN-TL model were 71.1%, 70.5%, 73.4%, 60.6%, and 59.5%. The percentages of all ROIs improved. Overall, the augmented visual encoding model is indeed more inclined to select stronger invariant features.

#### 3.2.2. The Impact of Data Augmentation on the Encoding Performance for Each ROI

**Figure 6:**
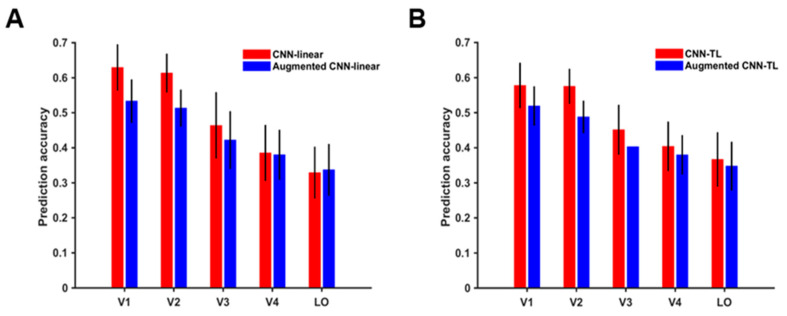
Prediction accuracies of visual encoding models for the ventral visual stream. (**A**) Prediction accuracies of the CNN-linear model and augmented CNN-linear model. The red column and blue column show the prediction accuracies of the CNN-linear model and the augmented CNN-linear model for a single ROI respectively. (**B**) Prediction accuracies of the CNN-TL model and augmented CNN-TL model. The red column and blue column show the prediction accuracies of the CNN-TL model and the augmented CNN-TL model for a single ROI, respectively.

According to prediction accuracy before and after data augmentation, the top 200 visual cortex voxels of response were studied, and the mean and variance of their prediction accuracies were drawn. The results of the CNN-linear model and CNN-TL model are shown in Figure 6A,B respectively. Except for the V4 and LO encoded by the CNN-linear model, the prediction accuracies of the data augmented visual encoding model for voxel responses of each ROI was significantly lower than that before the data augmentation (*t*-test, *p* < 0.05). These results resulted from training augmented encoding models without corresponding fMRI responses of augmented images. After the data augmentation, the average prediction accuracy of the CNN-linear model for each ROI decreased by 0.096, 0.100, 0.042, 0.005, and −0.008 respectively, and the average prediction accuracy of the CNN-TL model decreased by 0.058, 0.087, and 0.048, 0.024, and 0.0192 respectively. It is indicated that the average prediction accuracies of low-level visual areas decline more than those of high-level visual areas. This result reveals that voxels in low-level visual areas mainly respond to equivariant features, and voxels in high-level visual areas represent understanding to more invariant features.

#### 3.2.3. The Percentages of the Voxels Accurately Predicted in Each ROI

**Figure 7:**
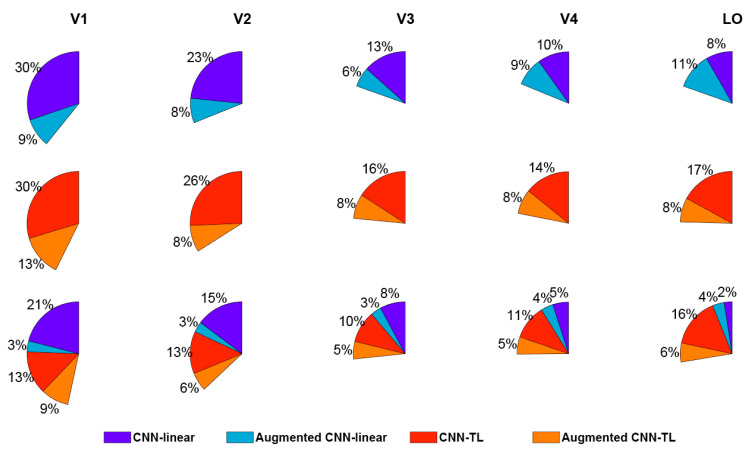
The advantage comparisons of visual encoding models. Each sector shows the proportion of voxels in one ROI, which can be predicted correctly by encoding models for comparison. Each color within sectors indicates the proportion of voxels best predicted by one encoding model. The CNN-linear model, augmented CNN-linear model, CNN-TL model, and augmented CNN-TL model are displayed by dark blue, light blue, red, and orange respectively.

According to the above results, the augmented encoding models prefer to select more invariant features in the CNN to encode visual voxels. Therefore, the augmented encoding models can more accurately predict responses of voxels with invariant feature representation in the human visual cortex. Then the invariant feature representation in each visual ROI can be studied through analyzing the distribution of voxels most accurately predicted by original encoding models and augmented encoding models. Firstly, all voxels that can be accurately predicted were counted. Then, the proportions of voxels more predicted by the original model and the enhanced model separately were calculated. The results of the CNN-linear model and the CNN-TL model are shown in the first two rows of Figure 7. Taking the last pie chart in the first row of the figure as an example, it shows that the CNN-linear encoding model and the augmented CNN-linear encoding model can accurately predict 19% of voxels in LO. Among them, 8% of voxels were better predicted by the CNN-linear encoding model, and 11% were better predicted by the augmented CNN-linear encoding model. It is demonstrated that the proportion of voxels better predicted by the augmented models rises as the visual cortex transits from the low-level area to the high-level area. Especially in the LO of the CNN-linear model, the proportion was more than one half. These reveal that the invariant feature representations of high-level visual areas are stronger than low-level visual areas. However, some voxels in each ROI of the visual cortex were better predicted by the augmented encoding models, indicating that there are invariant feature representations in the entire visual information processing process.

The encoding results of the CNN-linear model and the augmented CNN-linear model, the CNN-TL model, and the augmented CNN-TL model were compared together as shown in the third row of Figure 7. For example, the first pie chart shows that 46% of voxels in V1 can be accurately predicted by those four models altogether, and 21%, 3%, 13%, and 9% of voxels are best predicted by the CNN-linear model, the augmented CNN-linear model, the CNN-TL model, and the augmented CNN-TL model, respectively. Compared with CNN-linear models, CNN-TL models have better encoding performance wholly except for V1. The advantage is swiftly enlarged from low-level visual areas to high-level visual areas. It is revealed that the non-linear mapping from CNN features to brain visual responses improves the encoding performance for high-level visual ROIs more than selecting invariant features from CNN features. On the whole, no matter what kind of encoding model, the encoding performance for the high-level visual areas is significantly weaker than that for the low-level visual areas.

## 4. Discussion

### 4.1. The Proposed Research Framework

There have always been many convoluted image-based visual tasks to settle in computer vision [49,50,51], such as image retrieval, image classification, semantic segmentation, image captioning, etc. However, the emergence of innovative computational models and learning algorithms has provided new approaches for solving those highly demanding tasks. CNNs have been proposed to be innovative and powerful access in recent years. As a forceful solution for plenty of complicated problems, CNNs have not only obtained success in computer vision but also catch the eyes of researchers in the field of psychology and neuroscience. It is commonly accepted that representation invariance plays a very important role in the success of CNNs. The same property exists in the human visual information processing process, which has been studied for several decades. However, the human visual system and CNNs are so sophisticated that there has been abounding confusion. Here, we proposed the research framework where we simultaneously explored the representation invariance mechanisms of CNNs and human visual information processing using the same dataset based on data augmentation technology. We compared similarities of the features in corresponding intermediate layers of Alexnet before and after data augmentation. For the human ventral visual stream, we studied several key questions for understanding the representation invariance mechanism based on fMRI encoding models, including the feature selection preference of encoding models, the impact of data augmentation on the encoding performance, and the percentages of the voxels accurately predicted in each ROI before and after data augmentation. Although there is considerable literature about the representation invariance of CNNs [30,31,32,33,34,35] and the human visual information processing process [36,37,38,39], few literature studies the two systems simultaneously. The research framework we proposed investigated the two complex systems under common conditions and explored their relationship. Finally, our results revealed the internal correlation in representation invariances of CNNs and human visual processing.

### 4.2. Network Architecture Is an Important Factor for the Representation Invariance of CNNs

Alexnet classified or recognized objects with high accuracy due to its robustness for some transformations [22]. This property can be explained by the high feature similarity of the last layer (see Figure 4), although input data was flipped, cropped, scaled, and so on. On the contrary, the feature similarities were low at later convolutional layers, indicating that the feature maps of intermediate layers in CNNs are sensitive to global transformations of the input images, similar to the conclusion drawn in [52]. Therefore, it might be speculated that the pooling operation and weight sharing in convolutional layers have limited importance in extracting invariant features of the input data. The feature similarities increasingly rose in the last three fully connected layers. Therefore, our results demonstrate that the overall architecture of CNNs, and the combination of convolution layers and fully connected layers, played a major role in transform-invariant representation, and then the former is responsible for detecting significant features and the later for feature integration analysis. Kheradpisheh et al. also found an interesting result that networks with deeper structure and convolutional layers with small filter size but with more feature planes could powerfully outperform all other models for invariant object recognition [16]. Hence only a very deep hierarchy network can invariantly represent large global transformations, considering pooling and convolution with the ordinarily small local space. Therefore, CNNs have been developing toward deeper architecture to achieve higher performances in complicated vision tasks.

### 4.3. The Representation Invariance of Human Visual Processing

The human brain visual system is so complex that representation invariance is just the tip of the iceberg. Only specific properties, like representation invariance, are studied thoroughly, can we understand the human brain more clearly. It was commonly accepted that there is representation invariance just in the high-level visual cortex, such as V4 [15,41,43] and IT [53]. However, our results suggested that the feature representation invariance displayed in the whole process of the ventral visual stream, according to more voxels better encoded by the augmented models in each ROI (see Figure 7). The larger deterioration of prediction accuracies in low-level areas (see Figure 6) and the higher proportion of voxels better predicted after data augmentation (see Figure 7) indicate that high-level visual areas have a completely stronger invariance in feature representation than low-level visual areas. This property has a forceful relationship with the responsibilities of each visual cortex in that the primary visual areas process more low-level features like textures and the senior visual areas process more low-level features like semantics. However, all areas except V1 respond more intensively to invariant features after data augmentation following the preference for more transform-invariant layers of augmented encoding models (see Figure 5).

### 4.4. The Interaction between CNNs and the Human Brain Visual System

The results of studies for representation invariance of CNNs and the human visual processing process have internal consistency, although they seem independent. Representation invariance of CNNs greatly benefits from the architecture, motivated by this feed-forward information flow and the hierarchical organization of the ventral visual stream, where representation invariance penetrates wholly. Naturally, the transform-invariant property of CNNs is far weaker than that of the human visual processing process, owing to the CNNs’ lack of significant representing mechanisms that exist in the human visual system. For example, CNNs are single feed-forward systems without any feedback mechanisms from later to earlier layers, which are considered to play a pivotal role in complex visual tasks [16]. Therefore, how to learn feedback connections in CNNs becomes a demanding problem to settle in the field of AI.

Brain vision research has played a guiding role in the development of CNNs. Lenet-5 [54], the first CNN, was proposed by LeCun inspired by the hierarchical architecture of the ventral visual stream, which was successfully applied to handwritten character recognition. After decades of development, Alexnet [22], a deeper network, made great achievements in Imagenet 2012, promoting the application craze of CNNs in various image-based vision tasks. Studies for object invariant recognition also obtained many inspirations from the human visual information processing process. The VisNet [55,56], a biologically plausible approach, was specifically constructed for invariant visual object recognition. The neurophysiological and computational model taken here concentrated on a feature hierarchy architecture, which was like the ventral visual stream from V1 to TE. Based on the statistics of the input images, the model built invariant representations by self-organizing learning. Moreover, the hierarchical systems had many neurophysiological processes including short-term memory and associative learning of the rewarding and punishing properties, increasingly improving the performance in object recognition.

As powerful computing models, CNNs have been used for modeling the primate visual system in recent years. Agrawal et al. first encoded responses of the ventral visual cortex using CNN features, remarkably enhancing prediction accuracy of visual voxels [1]. Based on a 96-image dataset, Khaligh-Razavi et al. compared the representational geometry of neuronal recordings in IT areas of humans and monkeys with several computational models, including one CNN, showing that IT can be represented by supervised CNNs [57]. More specifically, Güçlü et al. mapped disparate CNN intermediate layers onto the ventral visual cortex and computed the response similarities between CNNs and the fMRI data from different ROIs in the ventral visual stream [2]. With the development of CNNs, new network architecture, such as ResNet, was implemented for modeling the human visual information processing process, achieving better performance [58]. However, no matter which encoding model was used, including the CNN-TL model and the CNN-linear model, the encoding performance for higher-level visual areas was more deeply weakened than that for lower-level visual areas (see Figure 6). This demonstrates that the visual encoding model based on CNN features does not have enough ability to sufficiently represent higher-level visual areas, revealing the deep gap between CNNs and the human visual system in higher-level semantic understanding.

### 4.5. Future Work

Our study on representation invariance of CNNs and the human visual information processing process not only verified the pivotal role of CNNs architecture in transform-invariant representation but also found that representation invariance exists in the whole ventral visual stream. Hence, the internal consistency of representation invariance of the two systems was identified, thoroughly showing the advantages of studying the two perplexing systems together. However, there are several elements to further develop in this study. Due to the limitation of fMRI experiments, a mimic of the human brain, as study object, replaced the real human brain. If the experimental conditions permit, the next step is to collect real fMRI signals of the human brain viewing data augmented image database for analysis. Moreover, other transformation invariances to specific parameters, such as shape, texture, color, etc., should be further investigated, facilitating deeper understanding of CNNs and the human brain. Another tricky problem is the absence of standard methods for assessing representation invariance. Therefore, significant and urgent work for both neuroscientists and computational modelers is to set a standard and robust proceeding for quantifying representation invariances of CNNs and the human visual information processing process.

## Data Availability

The detailed information about the fMRI data is provided in previous studies, and the public dataset can be downloaded from http://crcns.org/data-sets/vc/vim-1.
